# CSF glial markers are elevated in a subset of patients with genetic frontotemporal dementia

**DOI:** 10.1002/acn3.51672

**Published:** 2022-10-17

**Authors:** Ione O. C. Woollacott, Imogen J. Swift, Aitana Sogorb‐Esteve, Carolin Heller, Kathryn Knowles, Arabella Bouzigues, Lucy L. Russell, Georgia Peakman, Caroline V. Greaves, Rhian Convery, Amanda Heslegrave, James B. Rowe, Barbara Borroni, Daniela Galimberti, Pietro Tiraboschi, Mario Masellis, Maria Carmela Tartaglia, Elizabeth Finger, John C. van Swieten, Harro Seelaar, Lize Jiskoot, Sandro Sorbi, Chris R. Butler, Caroline Graff, Alexander Gerhard, Robert Laforce, Raquel Sanchez‐Valle, Alexandre de Mendonça, Fermin Moreno, Matthis Synofzik, Rik Vandenberghe, Simon Ducharme, Isabelle Le Ber, Johannes Levin, Markus Otto, Florence Pasquier, Isabel Santana, Henrik Zetterberg, Jonathan D. Rohrer, the Genetic FTD Initiative, GENFI, Annabel Nelson, Martina Bocchetta, David Cash, David L. Thomas, Emily Todd, Hanya Benotmane, Jennifer Nicholas, Kiran Samra, Rachelle Shafei, Carolyn Timberlake, Thomas Cope, Timothy Rittman, Alberto Benussi, Enrico Premi, Roberto Gasparotti, Silvana Archetti, Stefano Gazzina, Valentina Cantoni, Andrea Arighi, Chiara Fenoglio, Elio Scarpini, Giorgio Fumagalli, Vittoria Borracci, Giacomina Rossi, Giorgio Giaccone, Giuseppe Di Fede, Paola Caroppo, Sara Prioni, Veronica Redaelli, David Tang‐Wai, Ekaterina Rogaeva, Miguel Castelo‐Branco, Morris Freedman, Ron Keren, Sandra Black, Sara Mitchell, Christen Shoesmith, Robart Bartha, Rosa Rademakers, Jackie Poos, Janne M. Papma, Lucia Giannini, Rick van Minkelen, Yolande Pijnenburg, Benedetta Nacmias, Camilla Ferrari, Cristina Polito, Gemma Lombardi, Valentina Bessi, Michele Veldsman, Christin Andersson, Hakan Thonberg, Linn Öijerstedt, Vesna Jelic, Paul Thompson, Tobias Langheinrich, Albert Lladó, Anna Antonell, Jaume Olives, Mircea Balasa, Nuria Bargalló, Sergi Borrego‐Ecija, Ana Verdelho, Carolina Maruta, Catarina B. Ferreira, Gabriel Miltenberger, Frederico Simões do Couto, Alazne Gabilondo, Ana Gorostidi, Jorge Villanua, Marta Cañada, Mikel Tainta, Miren Zulaica, Myriam Barandiaran, Patricia Alves, Benjamin Bender, Carlo Wilke, Lisa Graf, Annick Vogels, Mathieu Vandenbulcke, Philip Van Damme, Rose Bruffaerts, Koen Poesen, Pedro Rosa‐Neto, Serge Gauthier, Agnès Camuzat, Alexis Brice, Anne Bertrand, Aurélie Funkiewiez, Daisy Rinaldi, Dario Saracino, Olivier Colliot, Sabrina Sayah, Catharina Prix, Elisabeth Wlasich, Olivia Wagemann, Sandra Loosli, Sonja Schönecker, Tobias Hoegen, Jolina Lombardi, Sarah Anderl‐Straub, Adeline Rollin, Gregory Kuchcinski, Maxime Bertoux, Thibaud Lebouvier, Vincent Deramecourt, Beatriz Santiago, Diana Duro, Maria João Leitão, Maria Rosario Almeida, Miguel Tábuas‐Pereira, Sónia Afonso

**Affiliations:** ^1^ Department of Neurodegenerative Disease, Dementia Research Centre UCL Institute of Neurology, Queen Square London United Kingdom; ^2^ UK Dementia Research Institute at UCL London United Kingdom; ^3^ Department of Clinical Neurosciences and Cambridge University Hospitals NHS Trust and Medical Research Council Cognition and Brain Sciences Unit University of Cambridge Cambridge United Kingdom; ^4^ Centre for Neurodegenerative Disorders, Department of Clinical and Experimental Sciences University of Brescia Brescia Italy; ^5^ Department of Biomedical, Surgical and Dental Sciences University of Milan Milan Italy; ^6^ Fondazione IRCCS Ca' Granda, Ospedale Maggiore Policlinico Milan Italy; ^7^ Fondazione IRCCS Istituto Neurologico Carlo Besta Milan Italy; ^8^ Sunnybrook Health Sciences Centre, Sunnybrook Research Institute University of Toronto Toronto Canada; ^9^ Tanz Centre for Research in Neurodegenerative Diseases University of Toronto Toronto Canada; ^10^ Department of Clinical Neurological Sciences University of Western Ontario London Ontario Canada; ^11^ Department of Neurology Erasmus Medical Centre Rotterdam The Netherlands; ^12^ Department of Neurofarba University of Florence Florence Italy; ^13^ IRCCS Fondazione Don Carlo Gnocchi Florence Italy; ^14^ Nuffield Department of Clinical Neurosciences, Medical Sciences Division University of Oxford Oxford United Kingdom; ^15^ Department of Brain Sciences Imperial College London United Kingdom; ^16^ Center for Alzheimer Research, Division of Neurogeriatrics, Department of Neurobiology Care Sciences and Society, Bioclinicum, Karolinska Institutet Solna Sweden; ^17^ Unit for Hereditary Dementias, Theme Aging Karolinska University Hospital Solna Sweden; ^18^ Division of Neuroscience and Experimental Psychology, Wolfson Molecular Imaging Centre University of Manchester Manchester United Kingdom; ^19^ Departments of Geriatric Medicine and Nuclear Medicine University of Duisburg‐Essen Essen Germany; ^20^ Cerebral Function Unit, Manchester Centre for Clinical Neurosciences Salford Royal NHS Foundation Trust Salford United Kingdom; ^21^ Clinique Interdisciplinaire de Mémoire, Département des Sciences Neurologiques, CHU de Québec, and Faculté de Médecine Université Laval Québec Canada; ^22^ Alzheimer's disease and Other Cognitive Disorders Unit, Neurology Service, Hospital Clínic, Institut d'Investigacións Biomèdiques August Pi I Sunyer University of Barcelona Barcelona Spain; ^23^ Faculty of Medicine University of Lisbon Lisbon Portugal; ^24^ Cognitive Disorders Unit, Department of Neurology Donostia University Hospital San Sebastian Gipuzkoa Spain; ^25^ Neuroscience Area Biodonostia Health Research Institute San Sebastian Gipuzkoa Spain; ^26^ Department of Neurodegenerative Diseases, Hertie‐Institute for Clinical Brain Research and Center of Neurology University of Tübingen Tübingen Germany; ^27^ Center for Neurodegenerative Diseases (DZNE) Tübingen Germany; ^28^ Laboratory for Cognitive Neurology, Department of Neurosciences KU Leuven Leuven Belgium; ^29^ Neurology Service University Hospitals Leuven Leuven Belgium; ^30^ Leuven Brain Institute, KU Leuven Leuven Belgium; ^31^ Douglas Mental Health University Institute, Department of Psychiatry McGill University Montreal Canada; ^32^ McConnell Brain Imaging Centre, Montreal Neurological Institute, Department of Neurology & Neurosurgery McGill University Montreal Canada; ^33^ Sorbonne Université, Paris Brain Institute – Institut du Cerveau – ICM, Inserm U1127, CNRS UMR 7225, AP‐HP ‐ Hôpital Pitié‐Salpêtrière Paris France; ^34^ Centre de référence des démences rares ou précoces, IM2A, Département de Neurologie AP‐HP ‐ Hôpital Pitié‐Salpêtrière Paris France; ^35^ Département de Neurologie AP‐HP ‐ Hôpital Pitié‐Salpêtrière Paris France; ^36^ Neurologische Klinik und Poliklinik, Ludwig‐Maximilians‐Universität Munich Germany; ^37^ Center for Neurodegenerative Diseases (DZNE) Munich Germany; ^38^ Munich Cluster of Systems Neurology Munich Germany; ^39^ Department of Neurology University of Ulm Ulm Germany; ^40^ Univ Lille Lille France; ^41^ Inserm 1172 Lille France; ^42^ CHU, CNR‐MAJ, Labex Distalz, LiCEND Lille Lille France; ^43^ Neurology Service, Faculty of Medicine University Hospital of Coimbra (HUC), University of Coimbra Coimbra Portugal; ^44^ Center for Neuroscience and Cell Biology, Faculty of Medicine University of Coimbra Coimbra Portugal; ^45^ Department of Psychiatry and Neurochemistry, Institute of Neuroscience and Physiology The Sahlgrenska Academy at the University of Gothenburg Mölndal Sweden; ^46^ Clinical Neurochemistry Laboratory Sahlgrenska University Hospital Mölndal Sweden; ^47^ Hong Kong Center for Neurodegenerative Diseases Clear Water Bay, Hong Kong China

## Abstract

**Background:**

Neuroinflammation has been shown to be an important pathophysiological disease mechanism in frontotemporal dementia (FTD). This includes activation of microglia, a process that can be measured in life through assaying different glia‐derived biomarkers in cerebrospinal fluid. However, only a few studies so far have taken place in FTD, and even fewer focusing on the genetic forms of FTD.

**Methods:**

We investigated the cerebrospinal fluid concentrations of TREM2, YKL‐40 and chitotriosidase using immunoassays in 183 participants from the Genetic FTD Initiative (GENFI) study: 49 *C9orf72* (36 presymptomatic, 13 symptomatic), 49 *GRN* (37 presymptomatic, 12 symptomatic) and 23 *MAPT* (16 presymptomatic, 7 symptomatic) mutation carriers and 62 mutation‐negative controls. Concentrations were compared between groups using a linear regression model adjusting for age and sex, with 95% bias‐corrected bootstrapped confidence intervals. Concentrations in each group were correlated with the Mini‐Mental State Examination (MMSE) score using non‐parametric partial correlations adjusting for age. Age‐adjusted *z*‐scores were also created for the concentration of markers in each participant, investigating how many had a value above the 95th percentile of controls.

**Results:**

Only chitotriosidase in symptomatic *GRN* mutation carriers had a concentration significantly higher than controls. No group had higher TREM2 or YKL‐40 concentrations than controls after adjusting for age and sex. There was a significant negative correlation of chitotriosidase concentration with MMSE in presymptomatic *GRN* mutation carriers. In the symptomatic groups, for TREM2 31% of *C9orf72*, 25% of *GRN*, and 14% of *MAPT* mutation carriers had a concentration above the 95^th^ percentile of controls. For YKL‐40 this was 8% *C9orf72*, 8% *GRN* and 0% *MAPT* mutation carriers, whilst for chitotriosidase it was 23% *C9orf72*, 50% *GRN*, and 29% *MAPT* mutation carriers.

**Conclusions:**

Although chitotriosidase concentrations in *GRN* mutation carriers were the only significantly raised glia‐derived biomarker as a group, a subset of mutation carriers in all three groups, particularly for chitotriosidase and TREM2, had elevated concentrations. Further work is required to understand the variability in concentrations and the extent of neuroinflammation across the genetic forms of FTD. However, the current findings suggest limited utility of these measures in forthcoming trials.

## Introduction

Frontotemporal dementia (FTD) is a neurodegenerative disorder that leads to progressive behavioral, linguistic and motor disturbances, often at a relatively young age. It is genetic in about a third of cases, with mutations in *C9orf72*, *GRN* and *MAPT* being the commonest causes.^1^ However, little is known about the underlying pathophysiological processes that occur in FTD. The study of asymptomatic at‐risk genetic FTD family members has provided a unique window into the pathogenesis and evolution of the disorder over time with deeply phenotyped cohort studies such as the Genetic FTD Initiative (GENFI) allowing in vivo analysis of biomarkers indicative of cellular dysfunction.^2^, ^3^, ^4^ An area of recent interest in FTD has been that of chronic neuroinflammation and glial dysfunction and how these processes contribute to disease.^5^, ^6^

Neuroinflammation is a complex and multistage process involving activation of specific cells such as microglia within the central nervous system and release of a series of pro‐ and anti‐inflammatory factors. Studies in FTD have recently started to focus on measuring microglial activation during life through cerebrospinal fluid (CSF) biomarkers. Such measures include soluble triggering receptor expressed on myeloid cells 2 (TREM2), which has been extensively investigated in Alzheimer's disease (AD),^7^, ^8^, ^9^ and the chitinases. This second group includes chitotriosidase (CHIT1) and YKL‐40 (otherwise known as chitinase‐3‐like protein 1 or CHI3L1). Investigation of each of these measures has so far shown mixed results, with some studies reporting higher levels and some suggesting that there are no differences from controls.^10^, ^11^, ^12^, ^13^, ^14^, ^15^, ^16^ The majority of these studies have examined an undifferentiated FTD cohort, not stratified by genetic or pathological subtype, but in the small studies that have investigated specific forms of FTD, there is some evidence for a particular role of microglial activation in those with progranulin (*GRN)* mutations^12^, ^16^ and those with associated amyotrophic lateral sclerosis (ALS).^14^

We therefore set out to establish whether levels of glia‐derived biomarkers vary according to the genetic FTD subtype, and also whether levels change presymptomatically in each genetic subtype, using CSF samples from the GENFI cohort.

## Methods

### Participants

Participants were recruited from the international multicentre GENFI study including sites in the United Kingdom, Canada, Sweden, Netherlands, Belgium, Spain, France, Portugal, Italy, and Germany. Ethical approval was obtained for the study, and all participants provided informed written consent. Participants underwent a standardised GENFI clinical assessment including a medical history, physical examination, and the Mini‐Mental State Examination (MMSE).

CSF samples were collected from participants at individual GENFI sites and then processed and stored at −80°C at each site according to a standardised GENFI protocol. CSF samples collected from participants at other external GENFI sites were transferred to University College London (UCL) at −80°C and on arrival were immediately stored at −80°C until being thawed on the day of the experiment.

In total, samples from 183 participants were used: 62 mutation‐negative controls and 121 mutation carriers. In the mutation carrier group there were 49 C9orf72 mutation carriers (36 presymptomatic and 13 symptomatic, all with behavioral variant FTD (bvFTD^17^), except 1 with FTD‐ALS^18^), 49 *GRN* mutation carriers (37 presymptomatic and 12 symptomatic, of whom 8 had bvFTD and 4 had primary progressive aphasia^19^), and 23 *MAPT* mutation carriers (16 presymptomatic and 7 symptomatic, all with bvFTD).

Immunoassays were used to measure concentrations of soluble TREM2, YKL‐40 and chitotriosidase as per below, with each assay carried out in duplicate by a single experimenter (IW) on all samples on the same day at UCL. Coefficients of variation were less than 10% for each assay.

CSF soluble TREM2 levels were measured using a previously published immunoassay^20^ on the Meso Scale Discovery (MSD) platform, with a biotinylated polyclonal goat anti‐human TREM2 capture antibody (0.25 μg/mL; BAF1828, R&D Systems, Minneapolis, MN, USA) and monoclonal mouse anti‐human TREM2 detection antibody (1 μg/mL; (B‐3): sc373828, Santa Cruz Biotechnology, Texas, USA). The two chitinase proteins were measured as follows: CSF YKL‐40 levels using the commercially available Human YKL‐40 Immunoassay Kit on the MSD platform and CSF chitotriosidase (CHIT1) levels using the commercially available CircuLex Human ELISA Kit (MBL International, Woburn, MA, USA). Coefficients of variation were less than 10% for each assay.

### Statistical analysis

Statistical analyses were performed using STATA Release 16. College Station, TX: StataCorp LLC. Sex and age were compared between groups using chi‐squared and *t*‐tests, respectively.

Concentrations of each of the glia‐derived biomarkers were compared between groups using a linear regression model adjusting for age and sex, with 95% bias‐corrected bootstrapped confidence intervals with 1000 repetitions (as data was non‐normally distributed).

The association of concentrations of each of the three biomarkers with the MMSE score was investigated in each genetic group by assessing non‐parametric partial correlations (adjusting for age).

Lastly, the association of concentrations of each the three biomarkers with age was assessed by performing a Spearman correlation with each measure in controls as well as looking at the mean and standard deviation concentration in each decade of life from the 20s to the 60s. These values were used to create an age‐adjusted *z*‐score for each participant in each measure. We then investigated how many individual participants had an abnormal *z*‐score, defined as being above the 95th centile (*z* = 1.65) of controls.

## Results

### Demographics

The presymptomatic *C9orf72, GRN*, and *MAPT* mutation carrier groups were not significantly different in sex or age to the control group, but each of the symptomatic groups contained more men and were older than the controls (*p* < 0.05 for each comparison).

### Microglial activation markers in each genetic group

**Figure 1:**
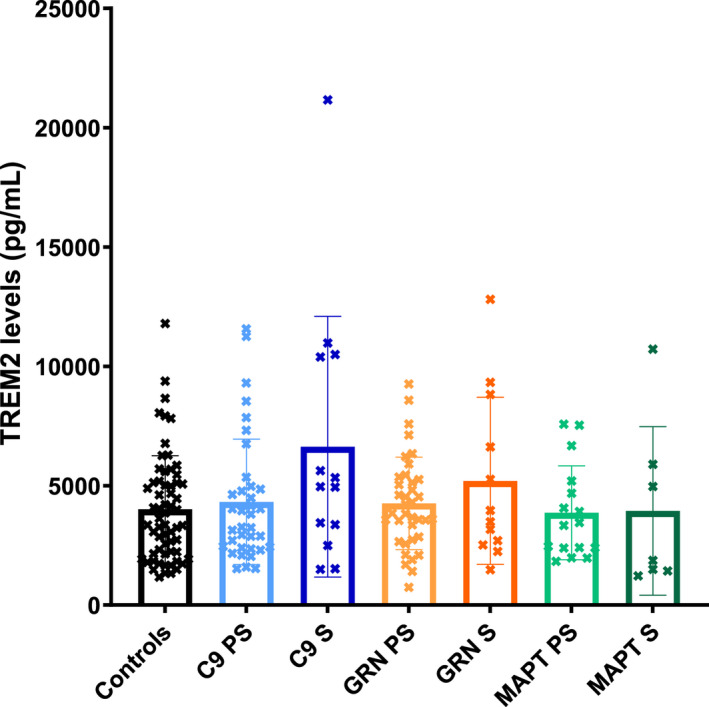
Mean concentrations within each group of TREM2. S = symptomatic, PS = presymptomatic.

**Figure 2:**
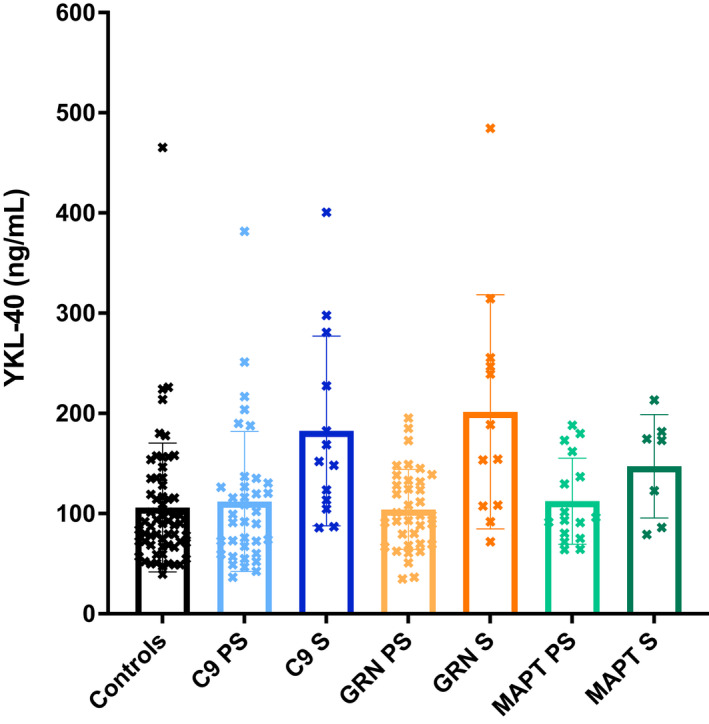
Mean concentrations within each group of YKL‐40. S = symptomatic, PS = presymptomatic.

**Figure 3:**
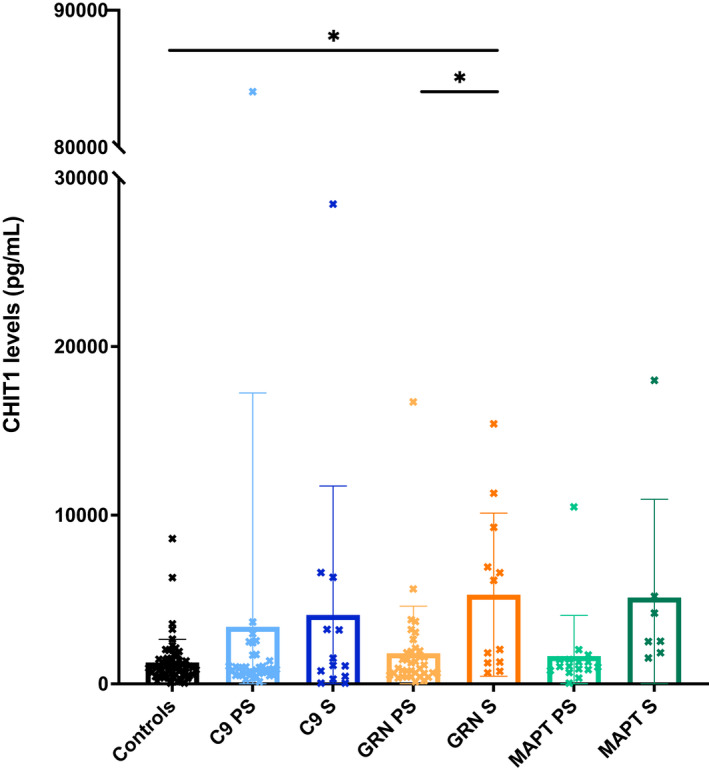
Mean concentrations within each group of CHIT1. S = symptomatic, PS = presymptomatic.

No significant differences were seen between groups for either TREM2 (Table 1; Fig. 1; Table S1A) or YKL‐40 (Table 1; Fig. 2; Table S1B). However, the chitotriosidase concentrations were higher in the symptomatic *GRN* mutation carriers compared with both the controls (adjusted mean difference 3683.5, 95% confidence intervals 776.5, 6590.5, *p* = 0.013) and presymptomatic *GRN* mutation carriers (3203.1, 95% CI 10.3, 6395.8, *p* = 0.049) (Table 1; Fig. 3; Table S1C).

**Table 1:** Demographic data showing the number of participants as well as the age, sex (percentage males) and education of each group.

	Control	*C9orf72* presymptomatic	*C9orf72* symptomatic	*GRN* presymptomatic	*GRN* symptomatic	*MAPT* presymptomatic	*MAPT* symptomatic
Number of participants	62	36	13	37	12	16	7
Sex (N and % of male in each group)	27 (43.5)	15 (41.7)	10 (76.9)	18 (48.6)	6 (50)	5 (31.3)	5 (71.4)
Age at CSF collection, years, mean (SD)	46.0 (13.2)	45.7 (11.2)	65.3 (8.5)	47.9 (12.5)	65.0 (6.1)	44.2 (10.4)	58.6 (6.7)
CSF TREM2, pg/mL, mean (SD)	4015.2 (2238.7)	4323.9 (2630.1)	6633.5 (5461.5)	4260.9 (1936.4)	5203.3 (3499.9)	3869.6 (1964.0)	3944.2 (3532.7)
CSF YKL‐40, ng/mL, mean (SD)	106.1 (64.2)	112.0 (69.8)	182.5 (94.7)	104.0 (39.9)	201.4 (116.9)	112.4 (43.0)	147.2 (51.6)
CSF CHIT1, pg/mL, mean (SD)	1268.8 (1367.1)	3388.9 (13852.8)	4083.2 (7649.5)	1811.4 (2792.2)	5288.2 (4829.6)	1645.2 (2408.5)	5113.6 (5828.6)

Five participants had undetectable levels of chitotriosidase in CSF, including on repeat testing. These were two symptomatic *C9orf72* mutation carriers, one asymptomatic *MAPT* mutation carrier and two controls. Approximately 6% of the population possess a homozygous 24 base pair duplication in exon 10 of the *CHIT1* gene, which leads to a complete enzymatic deficiency of chitotriosidase (Boot et al, 1998) and undetectable levels of chitotriosidase in CSF (Abu‐Rumeileh et al, 2019). These five individuals (2.7% cohort) were likely to be carriers of this mutation, given their undetectable levels. A repeat analysis excluding these cases did not affect the main results, with a significant difference still seen between symptomatic *GRN* mutation carriers and controls (adjusted mean difference 3525.0, 95% confidence intervals 553.6, 6496.3, *p* = 0.020).

### Correlation of microglial markers with cognition

The only significant (negative) correlation of the measures with MMSE was seen with chitotriosidase concentration in the presymptomatic *GRN* mutation carriers (*r* = −0.51, *p* = 0.0016, Table 2; Fig. S1).

**Table 2:** Partial correlations (adjusting for age) between microglial activation markers and cognition measured by the Mini‐Mental State Examination. *r* values are shown with *p*‐values below; significant correlations are shown in bold.

Genetic group	Genetic status	TREM2	YKL‐40	CHIT1
C9orf72	Presymptomatic	−0.03	−0.16	−0.11
		0.8282	0.3439	0.5161
	Symptomatic	0.09	−0.22	−0.16
		0.7969	0.5086	0.6849
GRN	Presymptomatic	−0.24	−0.27	**−0.51**
		0.1585	0.1199	**0.0016**
	Symptomatic	0.20	−0.48	−0.25
		0.6332	0.2272	0.5493
MAPT	Presymptomatic	−0.23	0.04	−0.28
		0.4287	0.8973	0.3484
	Symptomatic	−0.64	−0.37	−0.79
		0.2438	0.5384	0.1105

### Age‐adjusted *z*‐scores

Mean (standard deviation) concentrations of each of the markers in each of decade of life from 20 to 70 in the controls are shown in Table S2 along with the Spearman correlations of each measure with age: TREM2 *r* = 0.42, *p* = 0.0008, YKL‐40 *r* = 0.71, *p* < 0.0001, chitotriosidase *r* = 0.21, *p* = 0.1013.

In the symptomatic groups, for TREM2, 31% of *C9orf72* mutation carriers, 25% of *GRN* mutation carriers and 14% of *MAPT* mutation carriers had a concentration above the 95th percentile of controls (Table 3). Fewer presymptomatic participants had a high concentration but for the *C9orf72* (14%) and *MAPT* (13%) mutation groups the percentage of cases was above 5%.

**Table 3:** Percentage of participants in each group where the concentration of the microglial activation marker was higher than an age‐adjusted *z*‐score of 1.65.

Genetic group	Genetic status	TREM2	YKL‐40	CHIT1
*C9orf72*	Presymptomatic	14	8	8
	Symptomatic	31	8	23
*GRN*	Presymptomatic	5	0	11
	Symptomatic	25	8	50
*MAPT*	Presymptomatic	13	19	6
	Symptomatic	14	0	29

Only 8% of symptomatic *C9orf72* and *GRN* mutation carriers and none of the symptomatic *MAPT* mutation carriers had a concentration above the 95% centile for YKL‐40, with variable numbers in the presymptomatic mutation carriers.

However, for chitotriosidase 50% of the symptomatic *GRN* group, 29% of the *MAPT* group and 23% of the *C9orf72* group had a high concentration. As with the other measures, there were fewer cases with high concentrations in the presymptomatic group with 11% of *GRN*, 8% of *C9orf72*, and 6% of *MAPT* mutation carriers having a chitotriosidase level above the 95th percentile.

## Discussion

This study examined levels of three glia‐derived biomarkers, TREM2, YKL‐40 and chitotriosidase, in the CSF of people with genetic FTD due to mutations in *GRN, C9orf72*, and *MAPT*. On a group basis only chitotriosidase levels were raised, and only in the symptomatic *GRN* mutation carrier group. No changes were seen in the presymptomatic groups compared with controls. However in the presymptomatic *GRN* mutation carrier group there was a significant negative correlation with MMSE suggesting that chitotriosidases levels increase in proximity to symptom onset as cognition starts to become affected. Investigating age‐adjusted individual values of the biomarkers, concentrations are very variable in each presymptomatic and symptomatic genetic group, but there are higher proportions of people than expected with increased levels, particularly of TREM2 and chitotriosidase across all three symptomatic genetic groups.

There have been few other studies of glia‐derived biomarkers in genetic FTD.^12^, ^13^, ^14^, ^15^, ^16^, ^21^ An initial small study of CSF chitotriosidase^13^ found similar levels in genetic FTD to controls, but cohorts included a smaller number of cases and combined genetic subtypes into one group rather than investigating each subtype. In one further study that investigated specific genetic groups in small cohorts, increased chitotriosidase was seen only in *GRN* mutation carriers.^16^ This current study adds to these prior investigations by showing raised levels of chitotriosidase in *GRN* mutation carriers as a group but also higher levels in a subset of patients from the other genetic groups as well.

Raised chitotriosidase levels in *GRN* mutation carriers are consistent with multiple studies showing raised levels of other inflammatory markers in CSF or blood^3^, ^22^, ^23^, ^24^ and significant microglial dysfunction and activation in *GRN* mutation mouse models.^25^, ^26^, ^27^ *GRN* mutation models also display lipid accumulating microglia with extensive lysosomal dysfunction,^28^, ^29^ and lysosomal dysfunction is seen in human *GRN* mutation carriers.^30^, ^31^ This could alter delivery of proteins to the glial cell membrane or affect release into CSF. CSF chitotriosidase levels are highly elevated in the lysosomal storage disorder Gaucher's disease,^32^ where macrophages are chronically activated and lysosomes are overwhelmed by accumulation of the sphingolipid glucocerebroside. Raised chitotriosidase levels in *GRN* mutation carriers may therefore represent chronic lysosomal failure of microglia due to progranulin insufficiency and lipid mishandling.

Chitotriosidase may be released from glial cells once neurodegeneration develops, as a generalised protective response. Excessively activated or dysfunctional, degenerating (senescent) microglia (due to mutation‐related mechanisms) may lead to a sustained increase in release of these proteins. This is likely to occur presymptomatically in *GRN* mutation carriers where abnormalities can already be seen in MRI white matter hyperintensities,^33^ which are associated with astrocytic and microglial activation and dystrophy. Evidence from the significant negative correlation with cognition suggests a rise in chitotriosidase as symptom onset approaches.

YKL‐40, otherwise known as chitinase‐3‐like protein 1, falls within the same chitinase class of proteins as chitotriosidase. Its levels are raised in multiple acute and chronic neurological disorders including AD. While a small number of studies have shown raised levels in undifferentiated FTD cohorts,^10^, ^11^, ^12^, ^13^, ^14^, ^15^ there are fewer studies of particular pathogenetic forms. In those that have investigated specific groups, higher concentrations of YKL‐40 are found in those with associated ALS,^14^ and in one prior small study from our group, in people with *GRN* and *MAPT* mutations.^16^ However, in this study we did not find raised levels in any of the three genetic forms of FTD when studied as groups or even a substantially increased number of cases with higher concentrations in the age‐adjusted *z*‐score analysis. The reason for these differences are unclear, but it is likely that the different pathophysiological forms of FTD have differing neuroinflammatory responses and when an undifferentiated FTD cohort is studied, the presence of a difference between that group and controls will depend on the exact pathological composition of the group. Further work is needed in this area but it suggests that at least for genetic forms of FTD, CSF YKL‐40 is not an ideal candidate for measuring neuroinflammation in clinical trials.

Similarly, TREM2 levels were not raised in the group comparisons for any of the three genotypes. However, a subset of cases in each of the *C9orf72*, *GRN* and *MAPT* mutation groups had high concentrations. As TREM2 normally promotes microglial activation, proliferation, migration, and survival,^34^ raised TREM2 levels may be a normal, protective response, supporting microglia during early neurodegeneration, suggesting that the rise in a subset of mutation carriers may be stage‐dependent. Further work is needed to look at what factors might cause raised levels in some cases but not others, and whether there are factors that might even impair sTREM2 release, causing a failure of levels to rise appropriately (and therefore potentially reducing microglial survival and exacerbating neuronal dysfunction further).

In the *C9orf72* patient group, levels of all glia‐derived biomarkers were similar to controls as a group but on investigation of individual values a subset of patients had high concentrations. Certainly mouse models of *C9orf72* expansions demonstrate florid glial activation.^35^, ^36^ However, a recent study found that immune dysfunction and microglial activation in a *C9orf72* model vary widely according to the mouse gut microbiome.^37^ Variants in *TMEM106B* also modify effects of the *C9orf72* expansion by impacting lysosomal function.^38^ These mechanisms may underlie the wide variability in glia‐derived biomarker levels in the *C9orf72* group. Examination of the impact of environmental and genetic modifiers on neuroinflammatory biomarkers in a larger *C9orf72* cohort would be useful to explore this. Intriguingly, CSF chitotriosidase levels in those with *C9orf72* expansions and an ALS phenotype have been previously shown to be higher than those with an FTD phenotype^39^ and future studies examining the interaction of these features will be important.

Similarly to the *C9orf72* group, the symptomatic *MAPT* mutation carriers showed no differences as a group to controls for any of the three glia‐derived markers. However, for chitotriosidase 29% of symptomatic mutation carriers, and for TREM2 14% of symptomatic mutation carriers, had concentrations above the 95^th^ centile cutoff for controls. Certainly some previous non‐clinical studies have suggested a role for inflammation in *MAPT*‐associated FTD,^40^, ^41^ so it will be important to investigate other inflammatory biomarkers and whether the change in *MAPT* mutations is stage‐specific.

The positive association of TREM2 and YKL‐40 levels with age is consistent with previous studies in sporadic FTD and AD.^10^, ^42^, ^43^ Microglial activity increases with aging, which may augment release of TREM2 by microglia as a protective response to neuronal loss in aging individuals,^34^ and this may also be the case for YKL‐40. Chitotriosidase levels were not associated with age in any group, consistent with other studies of AD and FTD.^13^, ^14^ This suggests that the high CSF chitotriosidase levels in patients with *GRN* mutations represents excessive microglial activation or dysfunction related to the underlying mutation itself, or neurodegeneration, rather than aging. It also emphasises the importance of adjusting analyses of fluid biomarker levels for age when comparing groups of individuals with large age ranges, particularly when age independently affects the biological function of interest.

Limitations of the study include the small size of the patient subgroups once stratified which may have limited power to detect significant differences between groups. However, this is inherent to a rare disease like genetic FTD. Measurement of chitotriosidase is in part limited by the occurrence of people with undetectable levels (five participants in this cohort), likely due to mutations in *CHIT1* and future studies should take this into account. Longitudinal measurements of glia‐derived biomarkers in CSF will be helpful to investigate in the future, including in individuals who convert during the study, allowing analysis of the hypothesis that chitotriosidase levels change in proximity to symptom onset.

In summary, whilst CSF chitotriosidase was the most promising biomarker in this study as a potential in vivo measure of neuroinflammation in genetic FTD, particularly in those with *GRN* mutations, there is much variability within each genetic group. More work is needed to understand the reasons for that variability e.g. whether it is related to the specific stage of the disease, or whether there are inherent pathophysiological differences in the extent of the neuroinflammatory response in some mutation carriers in comparison to others. This variability may spell problems for their use in clinical trials. Although some mutation carriers have high concentrations, others have levels that overlap with controls i.e. there is little dynamic range for ‘lowering’ of a neuroinflammatory measure in a therapeutic trial when the concentration is already in the control range. Many of the proposed drugs for genetic FTD target neuroinflammation either directly or indirectly but the findings in this study suggest that we have not yet found the ideal measure of this important pathophysiological process for such trials.

## Conflicts of Interest

HZ has served at scientific advisory boards and/or as a consultant for Abbvie, Alector, Annexon, Artery Therapeutics, AZTherapies, CogRx, Denali, Eisai, Nervgen, Novo Nordisk, Pinteon Therapeutics, Red Abbey Labs, Passage Bio, Roche, Samumed, Siemens Healthineers, Triplet Therapeutics, and Wave, has given lectures in symposia sponsored by Cellectricon, Fujirebio, Alzecure, Biogen, and Roche, and is a co‐founder of Brain Biomarker Solutions in Gothenburg AB (BBS), which is a part of the GU Ventures Incubator Program. The other authors declare that they have no conflict of interest.

## Authors' Contributions

IOCW and JDR contributed to the study design and acquisition and, analysis of the samples. IOCW, JDR, and IS contributed to the statistical interpretation of the data as well as drafting and revising the manuscript. All other authors contributed to the acquisition of data and study coordination as well as helping to critically review and revise the manuscript.

## Consent to Participate

All participants provided informed written consent prior to their inclusion.

## Supporting information


**Table S1** Adjusted mean differences, 95% bootstrapped confidence intervals, and *p*‐values from the linear regression models (adjusted for age and sex): (A) TREM2, (B) YKL‐40, (C) CHIT1. PS is presymptomatic, S is symptomatic.
**Table S2.** Mean (standard deviation) concentrations of the microglial activation markers in each decade of life within the controls (excluding the two undetectable concentrations of CHIT1 in controls). Spearman correlation of each measure with age was as follows: TREM2 *r* = 0.42, *p* = 0.0008, YKL‐40 *r* = 0.71, *p* < 0.0001, CHIT1 *r* = 0.21, *p* = 0.1013.
**Figure S1.** Partial correlations (adjusting for age) of CHIT1 with Mini‐Mental State Examination in GRN mutation carriers (A) presymptomatic and (B) symptomatic.

## Data Availability

Some GENFI data are available on reasonable request through application to the GENFI Data Access Committee.

## Appendix

List of GENFI consortium authors.

**Table:**

Author	Affiliation
Annabel Nelson	Department of Neurodegenerative Disease, Dementia Research Centre, UCL Queen Square Institute of Neurology, London, UK
Martina Bocchetta	Department of Neurodegenerative Disease, Dementia Research Centre, UCL Queen Square Institute of Neurology, London, UK
David Cash	Department of Neurodegenerative Disease, Dementia Research Centre, UCL Queen Square Institute of Neurology, London, UK
David L. Thomas	Neuroimaging Analysis Centre, Department of Brain Repair and Rehabilitation, UCL Institute of Neurology, Queen Square, London, UK
Emily Todd	Department of Neurodegenerative Disease, Dementia Research Centre, UCL Queen Square Institute of Neurology, London, UK
Hanya Benotmane	UK Dementia Research Institute at University College London, UCL Queen Square Institute of Neurology, London, UK
Jennifer Nicholas	Department of Medical Statistics, London School of Hygiene and Tropical Medicine, London, UK
Kiran Samra	Department of Neurodegenerative Disease, Dementia Research Centre, UCL Queen Square Institute of Neurology, London, UK
Rachelle Shafei	Department of Neurodegenerative Disease, Dementia Research Centre, UCL Queen Square Institute of Neurology, London, UK
Carolyn Timberlake	Department of Clinical Neurosciences, University of Cambridge, Cambridge, UK
Thomas Cope	Department of Clinical Neuroscience, University of Cambridge, Cambridge, UK
Timothy Rittman	Department of Clinical Neurosciences, University of Cambridge, Cambridge, UK
Alberto Benussi	Centre for Neurodegenerative Disorders, Department of Clinical and Experimental Sciences, University of Brescia, Brescia, Italy
Enrico Premi	Stroke Unit, ASST Brescia Hospital, Brescia, Italy
Roberto Gasparotti	Neuroradiology Unit, University of Brescia, Brescia, Italy
Silvana Archetti	Biotechnology Laboratory, Department of Diagnostics, ASST Brescia Hospital, Brescia, Italy
Stefano Gazzina	Neurology, ASST Brescia Hospital, Brescia, Italy
Valentina Cantoni	Centre for Neurodegenerative Disorders, Department of Clinical and Experimental Sciences, University of Brescia, Brescia, Italy
Andrea Arighi	Fondazione IRCCS Ca′ Granda Ospedale Maggiore Policlinico, Neurodegenerative Diseases Unit, Milan, Italy; University of Milan, Centro Dino Ferrari, Milan, Italy
Chiara Fenoglio	Fondazione IRCCS Ca′ Granda Ospedale Maggiore Policlinico, Neurodegenerative Diseases Unit, Milan, Italy; University of Milan, Centro Dino Ferrari, Milan, Italy
Elio Scarpini	Fondazione IRCCS Ca′ Granda Ospedale Maggiore Policlinico, Neurodegenerative Diseases Unit, Milan, Italy; University of Milan, Centro Dino Ferrari, Milan, Italy
Giorgio Fumagalli	Fondazione IRCCS Ca′ Granda Ospedale Maggiore Policlinico, Neurodegenerative Diseases Unit, Milan, Italy; University of Milan, Centro Dino Ferrari, Milan, Italy
Vittoria Borracci	Fondazione IRCCS Ca′ Granda Ospedale Maggiore Policlinico, Neurodegenerative Diseases Unit, Milan, Italy; University of Milan, Centro Dino Ferrari, Milan, Italy
Giacomina Rossi	Fondazione IRCCS Istituto Neurologico Carlo Besta, Milano, Italy
Giorgio Giaccone	Fondazione IRCCS Istituto Neurologico Carlo Besta, Milano, Italy
Giuseppe Di Fede	Fondazione IRCCS Istituto Neurologico Carlo Besta, Milano, Italy
Paola Caroppo	Fondazione IRCCS Istituto Neurologico Carlo Besta, Milano, Italy
Pietro Tiraboschi	Fondazione IRCCS Istituto Neurologico Carlo Besta, Milano, Italy
Sara Prioni	Fondazione IRCCS Istituto Neurologico Carlo Besta, Milano, Italy
Veronica Redaelli	Fondazione IRCCS Istituto Neurologico Carlo Besta, Milano, Italy
David Tang‐Wai	The University Health Network, Krembil Research Institute, Toronto, Canada
Ekaterina Rogaeva	Tanz Centre for Research in Neurodegenerative Diseases, University of Toronto, Toronto, Canada
Miguel Castelo‐Branco	Faculty of Medicine, University of Coimbra, Coimbra, Portugal
Morris Freedman	Baycrest Health Sciences, Rotman Research Institute, University of Toronto, Toronto, Canada
Ron Keren	The University Health Network, Toronto Rehabilitation Institute, Toronto, Canada
Sandra Black	Sunnybrook Health Sciences Centre, Sunnybrook Research Institute, University of Toronto, Toronto, Canada
Sara Mitchell	Sunnybrook Health Sciences Centre, Sunnybrook Research Institute, University of Toronto, Toronto, Canada
Christen Shoesmith	Department of Clinical Neurological Sciences, University of Western Ontario, London, Ontario, Canada
Robart Bartha	Department of Medical Biophysics, The University of Western Ontario, London, Ontario, Canada; Centre for Functional and Metabolic Mapping, Robarts Research Institute, The University of Western Ontario, London, Ontario, Canada
Rosa Rademakers	Center for Molecular Neurology, University of Antwerp
Jackie Poos	Department of Neurology, Erasmus Medical Center, Rotterdam, Netherlands
Janne M. Papma	Department of Neurology, Erasmus Medical Center, Rotterdam, Netherlands
Lucia Giannini	Department of Neurology, Erasmus Medical Center, Rotterdam, Netherlands
Rick van Minkelen	Department of Clinical Genetics, Erasmus Medical Center, Rotterdam, Netherlands
Yolande Pijnenburg	Amsterdam University Medical Centre, Amsterdam VUmc, Amsterdam, Netherlands
Benedetta Nacmias	Department of Neuroscience, Psychology, Drug Research and Child Health, University of Florence, Florence, Italy
Camilla Ferrari	Department of Neuroscience, Psychology, Drug Research and Child Health, University of Florence, Florence, Italy
Cristina Polito	Department of Biomedical, Experimental and Clinical Sciences “Mario Serio”, Nuclear Medicine Unit, University of Florence, Florence, Italy
Gemma Lombardi	Department of Neuroscience, Psychology, Drug Research and Child Health, University of Florence, Florence, Italy
Valentina Bessi	Department of Neuroscience, Psychology, Drug Research and Child Health, University of Florence, Florence, Italy
Michele Veldsman	Nuffield Department of Clinical Neurosciences, Medical Sciences Division, University of Oxford, Oxford, UK
Christin Andersson	Department of Clinical Neuroscience, Karolinska Institutet, Stockholm, Sweden
Hakan Thonberg	Center for Alzheimer Research, Division of Neurogeriatrics, Karolinska Institutet, Stockholm, Sweden
Linn Öijerstedt	Center for Alzheimer Research, Division of Neurogeriatrics, Department of Neurobiology, Care Sciences and Society, Bioclinicum, Karolinska Institutet, Solna, Sweden; Unit for Hereditary Dementias, Theme Aging, Karolinska University Hospital, Solna, Sweden
Vesna Jelic	Division of Clinical Geriatrics, Karolinska Institutet, Stockholm, Sweden
Paul Thompson	Division of Neuroscience and Experimental Psychology, Wolfson Molecular Imaging Centre, University of Manchester, Manchester, UK
Tobias Langheinrich	Division of Neuroscience and Experimental Psychology, Wolfson Molecular Imaging Centre, University of Manchester, Manchester, UK; Manchester Centre for Clinical Neurosciences, Department of Neurology, Salford Royal NHS Foundation Trust, Manchester, UK
Albert Lladó	Alzheimer's disease and Other Cognitive Disorders Unit, Neurology Service, Hospital Clínic, Barcelona, Spain
Anna Antonell	Alzheimer's disease and Other Cognitive Disorders Unit, Neurology Service, Hospital Clínic, Barcelona, Spain
Jaume Olives	Alzheimer's disease and Other Cognitive Disorders Unit, Neurology Service, Hospital Clínic, Barcelona, Spain
Mircea Balasa	Alzheimer's disease and Other Cognitive Disorders Unit, Neurology Service, Hospital Clínic, Barcelona, Spain
Nuria Bargalló	Imaging Diagnostic Center, Hospital Clínic, Barcelona, Spain
Sergi Borrego‐Ecija	Alzheimer's disease and Other Cognitive Disorders Unit, Neurology Service, Hospital Clínic, Barcelona, Spain
Ana Verdelho	Department of Neurosciences and Mental Health, Centro Hospitalar Lisboa Norte – Hospital de Santa Maria & Faculty of Medicine, University of Lisbon, Lisbon, Portugal
Carolina Maruta	Laboratory of Language Research, Centro de Estudos Egas Moniz, Faculty of Medicine, University of Lisbon, Lisbon, Portugal
Catarina B. Ferreira	Laboratory of Neurosciences, Faculty of Medicine, University of Lisbon, Lisbon, Portugal
Gabriel Miltenberger	Faculty of Medicine, University of Lisbon, Lisbon, Portugal
Frederico Simões do Couto	Faculdade de Medicina, Universidade Católica Portuguesa
Alazne Gabilondo	Cognitive Disorders Unit, Department of Neurology, Donostia University Hospital, San Sebastian, Gipuzkoa, Spain; Neuroscience Area, Biodonostia Health Research Insitute, San Sebastian, Gipuzkoa, Spain
Ana Gorostidi	Neuroscience Area, Biodonostia Health Research Insitute, San Sebastian, Gipuzkoa, Spain
Jorge Villanua	OSATEK, University of Donostia, San Sebastian, Gipuzkoa, Spain
Marta Cañada	CITA Alzheimer, San Sebastian, Gipuzkoa, Spain
Mikel Tainta	Neuroscience Area, Biodonostia Health Research Insitute, San Sebastian, Gipuzkoa, Spain
Miren Zulaica	Neuroscience Area, Biodonostia Health Research Insitute, San Sebastian, Gipuzkoa, Spain
Myriam Barandiaran	Cognitive Disorders Unit, Department of Neurology, Donostia University Hospital, San Sebastian, Gipuzkoa, Spain; Neuroscience Area, Biodonostia Health Research Insitute, San Sebastian, Gipuzkoa, Spain
Patricia Alves	Neuroscience Area, Biodonostia Health Research Insitute, San Sebastian, Gipuzkoa, Spain; Department of Educational Psychology and Psychobiology, Faculty of Education, International University of La Rioja, Logroño, Spain
Benjamin Bender	Department of Diagnostic and Interventional Neuroradiology, University of Tübingen, Tübingen, Germany
Carlo Wilke	Department of Neurodegenerative Diseases, Hertie‐Institute for Clinical Brain Research and Center of Neurology, University of Tübingen, Tübingen, Germany; Center for Neurodegenerative Diseases (DZNE), Tübingen, Germany
Lisa Graf	Department of Neurodegenerative Diseases, Hertie‐Institute for Clinical Brain Research and Center of Neurology, University of Tübingen, Tübingen, Germany
Annick Vogels	Department of Human Genetics, KU Leuven, Leuven, Belgium
Mathieu Vandenbulcke	Geriatric Psychiatry Service, University Hospitals Leuven, Belgium; Neuropsychiatry, Department of Neurosciences, KU Leuven, Leuven, Belgium
Philip Van Damme	Neurology Service, University Hospitals Leuven, Belgium; Laboratory for Neurobiology, VIB‐KU Leuven Centre for Brain Research, Leuven, Belgium
Rose Bruffaerts	Department of Biomedical Sciences, University of Antwerp, Antwerp, Belgium; Biomedical Research Institute, Hasselt University, 3500 Hasselt, Belgium
Koen Poesen	Laboratory for Molecular Neurobiomarker Research, KU Leuven, Leuven, Belgium
Pedro Rosa‐Neto	Translational Neuroimaging Laboratory, McGill Centre for Studies in Aging, McGill University, Montreal, Québec, Canada
Serge Gauthier	Alzheimer Disease Research Unit, McGill Centre for Studies in Aging, Department of Neurology & Neurosurgery, McGill University, Montreal, Québec, Canada
Agnès Camuzat	Sorbonne Université, Paris Brain Institute – Institut du Cerveau – ICM, Inserm U1127, CNRS UMR 7225, AP‐HP – Hôpital Pitié‐Salpêtrière, Paris, France
Alexis Brice	Sorbonne Université, Paris Brain Institute – Institut du Cerveau – ICM, Inserm U1127, CNRS UMR 7225, AP‐HP – Hôpital Pitié‐Salpêtrière, Paris, France; Reference Network for Rare Neurological Diseases (ERN‐RND)
Anne Bertrand	Sorbonne Université, Paris Brain Institute – Institut du Cerveau – ICM, Inserm U1127, CNRS UMR 7225, AP‐HP – Hôpital Pitié‐Salpêtrière, Paris, France; Inria, Aramis project‐team, F‐75013, Paris, France; Centre pour l'Acquisition et le Traitement des Images, Institut du Cerveau et la Moelle, Paris, France
Aurélie Funkiewiez	Centre de référence des démences rares ou précoces, IM2A, Département de Neurologie, AP‐HP – Hôpital Pitié‐Salpêtrière, Paris, France; Sorbonne Université, Paris Brain Institute – Institut du Cerveau – ICM, Inserm U1127, CNRS UMR 7225, AP‐HP – Hôpital Pitié‐Salpêtrière, Paris, France
Daisy Rinaldi	Centre de référence des démences rares ou précoces, IM2A, Département de Neurologie, AP‐HP – Hôpital Pitié‐Salpêtrière, Paris, France; Sorbonne Université, Paris Brain Institute – Institut du Cerveau – ICM, Inserm U1127, CNRS UMR 7225, AP‐HP – Hôpital Pitié‐Salpêtrière, Paris, France; Département de Neurologie, AP‐HP – Hôpital Pitié‐Salpêtrière, Paris, France
Dario Saracino	Sorbonne Université, Paris Brain Institute – Institut du Cerveau – ICM, Inserm U1127, CNRS UMR 7225, AP‐HP – Hôpital Pitié‐Salpêtrière, Paris, France; Inria, Aramis project‐team, F‐75013, Paris, France; Centre de référence des démences rares ou précoces, IM2A, Département de Neurologie, AP‐HP – Hôpital Pitié‐Salpêtrière, Paris, France
Olivier Colliot	Sorbonne Université, Paris Brain Institute – Institut du Cerveau – ICM, Inserm U1127, CNRS UMR 7225, AP‐HP – Hôpital Pitié‐Salpêtrière, Paris, France; Inria, Aramis project‐team, F‐75013, Paris, France; Centre pour l'Acquisition et le Traitement des Images, Institut du Cerveau et la Moelle, Paris, France
Sabrina Sayah	Sorbonne Université, Paris Brain Institute – Institut du Cerveau – ICM, Inserm U1127, CNRS UMR 7225, AP‐HP – Hôpital Pitié‐Salpêtrière, Paris, France
Catharina Prix	Neurologische Klinik, Ludwig‐Maximilians‐Universität München, Munich, Germany
Elisabeth Wlasich	Neurologische Klinik, Ludwig‐Maximilians‐Universität München, Munich, Germany
Olivia Wagemann	Neurologische Klinik, Ludwig‐Maximilians‐Universität München, Munich, Germany
Sandra Loosli	Neurologische Klinik, Ludwig‐Maximilians‐Universität München, Munich, Germany
Sonja Schönecker	Neurologische Klinik, Ludwig‐Maximilians‐Universität München, Munich, Germany
Tobias Hoegen	Neurologische Klinik, Ludwig‐Maximilians‐Universität München, Munich, Germany
Jolina Lombardi	Department of Neurology, University of Ulm, Ulm
Sarah Anderl‐Straub	Department of Neurology, University of Ulm, Ulm, Germany
Adeline Rollin	CHU, CNR‐MAJ, Labex Distalz, LiCEND Lille, France
Gregory Kuchcinski	Univ Lille, France; Inserm 1172, Lille, France; CHU, CNR‐MAJ, Labex Distalz, LiCEND Lille, France
Maxime Bertoux	Inserm 1172, Lille, France; CHU, CNR‐MAJ, Labex Distalz, LiCEND Lille, France
Thibaud Lebouvier	Univ Lille, France; Inserm 1172, Lille, France; CHU, CNR‐MAJ, Labex Distalz, LiCEND Lille, France
Vincent Deramecourt	Univ Lille, France; Inserm 1172, Lille, France; CHU, CNR‐MAJ, Labex Distalz, LiCEND Lille, France
Beatriz Santiago	Neurology Department, Centro Hospitalar e Universitario de Coimbra, Coimbra, Portugal
Diana Duro	Faculty of Medicine, University of Coimbra, Coimbra, Portugal
Maria João Leitão	Centre of Neurosciences and Cell Biology, Universidade de Coimbra, Coimbra, Portugal
Maria Rosario Almeida	Faculty of Medicine, University of Coimbra, Coimbra, Portugal
Miguel Tábuas‐Pereira	Neurology Department, Centro Hospitalar e Universitario de Coimbra, Coimbra, Portugal
Sónia Afonso	Instituto Ciencias Nucleares Aplicadas a Saude, Universidade de Coimbra, Coimbra, Portugal
