# Supplementary material for: CSF glial markers are elevated in a subset of patients with genetic frontotemporal dementia
Source: Ann Clin Transl Neurol. Author manuscript; Available in PMC 2022 Dec 3. (PMC9639635; doi:10.1002/acn3.51672)
Supplement: Supporting Information [file EMS156476-supplement-Supporting_Information.pdf]

Supporting Information

Additional supporting information may be found online in the Supporting Information section at the end of the article.

Table S1 Adjusted mean differences, 95% bootstrapped confidence intervals, and *p*-values from the linear regression models (adjusted for age and sex): (A) TREM2, (B) YKL-40, (C) CHIT1. PS is presymptomatic, S is symptomatic.

Table S2. Mean (standard deviation) concentrations of the microglial activation markers in each decade of life within the controls (excluding the two undetectable concentrations of CHIT1 in controls). Spearman correlation of each measure with age was as follows: TREM2 $r = 0.42$, $p = 0.0008$, YKL-40 $r = 0.71$, $p < 0.0001$, CHIT1 $r = 0.21$, $p = 0.1013$.

Figure S1. Partial correlations (adjusting for age) of CHIT1 with Mini-Mental State Examination in GRN mutation carriers (A) presymptomatic and (B) symptomatic.

Appendix: List of GENFI consortium authors.

Author	Affiliation
Annabel Nelson	Department of Neurodegenerative Disease, Dementia Research Centre, UCL Queen Square Institute of Neurology, London, UK
Martina Bocchetta	Department of Neurodegenerative Disease, Dementia Research Centre, UCL Queen Square Institute of Neurology, London, UK
David Cash	Department of Neurodegenerative Disease, Dementia Research Centre, UCL Queen Square Institute of Neurology, London, UK
David L. Thomas	Neuroimaging Analysis Centre, Department of Brain Repair and Rehabilitation, UCL Institute of Neurology, Queen Square, London, UK
Emily Todd	Department of Neurodegenerative Disease, Dementia Research Centre, UCL Queen Square Institute of Neurology, London, UK
Hanya Benotmane	UK Dementia Research Institute at University College London, UCL Queen Square Institute of Neurology, London, UK
Jennifer Nicholas	Department of Medical Statistics, London School of Hygiene and Tropical Medicine, London, UK

(*Continued*)

Appendix Continued.

Author	Affiliation
Kiran Samra	Department of Neurodegenerative Disease, Dementia Research Centre, UCL Queen Square Institute of Neurology, London, UK
Rachelle Shafei	Department of Neurodegenerative Disease, Dementia Research Centre, UCL Queen Square Institute of Neurology, London, UK
Carolyn Timberlake	Department of Clinical Neurosciences, University of Cambridge, Cambridge, UK
Thomas Cope	Department of Clinical Neuroscience, University of Cambridge, Cambridge, UK
Timothy Rittman	Department of Clinical Neurosciences, University of Cambridge, Cambridge, UK
Alberto Benussi	Centre for Neurodegenerative Disorders, Department of Clinical and Experimental Sciences, University of Brescia, Brescia, Italy
Enrico Premi	Stroke Unit, ASST Brescia Hospital, Brescia, Italy
Roberto Gasparotti	Neuroradiology Unit, University of Brescia, Brescia, Italy
Silvana Archetti	Biotechnology Laboratory, Department of Diagnostics, ASST Brescia Hospital, Brescia, Italy
Stefano Gazzina	Neurology, ASST Brescia Hospital, Brescia, Italy
Valentina Cantoni	Centre for Neurodegenerative Disorders, Department of Clinical and Experimental Sciences, University of Brescia, Brescia, Italy
Andrea Arighi	Fondazione IRCCS Ca′ Granda Ospedale Maggiore Policlinico, Neurodegenerative Diseases Unit, Milan, Italy; University of Milan, Centro Dino Ferrari, Milan, Italy
Chiara Fenoglio	Fondazione IRCCS Ca′ Granda Ospedale Maggiore Policlinico, Neurodegenerative Diseases Unit, Milan, Italy; University of Milan, Centro Dino Ferrari, Milan, Italy
Elio Scarpini	Fondazione IRCCS Ca′ Granda Ospedale Maggiore Policlinico, Neurodegenerative Diseases Unit, Milan, Italy; University of Milan, Centro Dino Ferrari, Milan, Italy
Giorgio Fumagalli	Fondazione IRCCS Ca′ Granda Ospedale Maggiore Policlinico, Neurodegenerative Diseases Unit, Milan, Italy; University of Milan, Centro Dino Ferrari, Milan, Italy
Vittoria Borracci	Fondazione IRCCS Ca′ Granda Ospedale Maggiore Policlinico, Neurodegenerative Diseases Unit, Milan, Italy; University of Milan, Centro Dino Ferrari, Milan, Italy
Giacomina Rossi	Fondazione IRCCS Istituto Neurologico Carlo Besta, Milano, Italy
Giorgio Giaccone	Fondazione IRCCS Istituto Neurologico Carlo Besta, Milano, Italy
Giuseppe Di Fede	Fondazione IRCCS Istituto Neurologico Carlo Besta, Milano, Italy
Paola Caroppo	Fondazione IRCCS Istituto Neurologico Carlo Besta, Milano, Italy
Pietro Tiraboschi	Fondazione IRCCS Istituto Neurologico Carlo Besta, Milano, Italy

(*Continued*)

23289503, 0, Downloaded from https://onlinelibrary.wiley.com/doi/10.1002/acn3.51672 by Test, Wiley Online Library on [01/11/2022]. See the Terms and Conditions (https://onlinelibrary.wiley.com/terms-and-conditions) on Wiley Online Library for rules of use; OA articles are governed by the applicable Creative Commons License
