# Supplementary material for: CSF glial markers are elevated in a subset of patients with genetic frontotemporal dementia
Source: Ann Clin Transl Neurol. 2022 Oct 17;9(11):1764–77. doi: 10.1002/acn3.51672 (PMC9639635; doi:10.1002/acn3.51672)
Supplement: Supplementary file 1 [file ACN3-9-1764-s001.docx]

**Supplementary Table 1: Adjusted mean differences, 95% bootstrapped confidence intervals, and p-values from the linear regression models (adjusted for age and sex): a) TREM2, b) YKL-40, c) CHIT1. PS is presymptomatic, S is symptomatic.**

**a)**

|  | **C9 PS** |  | **C9 S** |  | **GRN PS** |  | **GRN S** |  | **MAPT PS** |  | **MAPT S** |  |
| --- | --- | --- | --- | --- | --- | --- | --- | --- | --- | --- | --- | --- |
| **Controls** | 334.1 |  | 1113.0 |  | 96.7 |  | -293.0 |  | -4.3 |  | -1059.3 |  |
|  | -585.1 | 1253.2 | -1790.9 | 4016.9 | -698.3 | 891.8 | -2364.9 | 1778.9 | -1109.1 | 1100.5 | -3775.3 | 1656.8 |
|  | 0.476 |  | 0.453 |  | 0.812 |  | 0.782 |  | 0.994 |  | 0.445 |  |
| **C9 PS** |  |  | 778.9 |  | -237.3 |  | -627.1 |  | -338.3 |  | -1393.3 |  |
|  |  |  | -2199.5 | 3757.4 | -1227.9 | 753.2 | -2823.0 | 1568.8 | -1564.0 | 887.4 | -4190.0 | 1403.3 |
|  |  |  | 0.608 |  | 0.639 |  | 0.576 |  | 0.588 |  | 0.329 |  |
| **C9 S** |  |  |  |  | -1016.3 |  | -1406.0 |  | -1117.3 |  | -2172.3 |  |
|  |  |  |  |  | -3907.2 | 1874.6 | -4817.1 | 2005.1 | -4196.9 | 1962.3 | -6017.0 | 1672.5 |
|  |  |  |  |  | 0.491 |  | 0.419 |  | 0.477 |  | 0.268 |  |
| **GRN PS** |  |  |  |  |  |  | -389.8 |  | -101.0 |  | -1156.0 |  |
|  |  |  |  |  |  |  | -2456.9 | 1677.3 | -1266.7 | 1064.7 | -3874.6 | 1562.5 |
|  |  |  |  |  |  |  | 0.712 |  | 0.865 |  | 0.405 |  |
| **GRN S** |  |  |  |  |  |  |  |  | 288.7 |  | -766.3 |  |
|  |  |  |  |  |  |  |  |  | -1989.6 | 2567.1 | -4030.4 | 2497.8 |
|  |  |  |  |  |  |  |  |  | 0.804 |  | 0.645 |  |
| **MAPT PS** |  |  |  |  |  |  |  |  |  |  | -1055.0 |  |
|  |  |  |  |  |  |  |  |  |  |  | -3908.0 | 1798.0 |
|  |  |  |  |  |  |  |  |  |  |  | 0.469 |  |
| **MAPT S** |  |  |  |  |  |  |  |  |  |  |  |  |
|  |  |  |  |  |  |  |  |  |  |  |  |  |
|  |  |  |  |  |  |  |  |  |  |  |  |  |

**b)**

|  | **C9 PS** |  | **C9 S** |  | **GRN PS** |  | **GRN S** |  | **MAPT PS** |  | **MAPT S** |  |
| --- | --- | --- | --- | --- | --- | --- | --- | --- | --- | --- | --- | --- |
| **Controls** | 6.9 |  | 19.6 |  | -7.2 |  | 39.5 |  | 11.6 |  | 3.8 |  |
|  | -17.0 | 30.8 | -27.8 | 66.9 | -23.8 | 9.3 | -26.3 | 105.3 | -14.4 | 37.6 | -34.6 | 42.2 |
|  | 0.572 |  | 0.417 |  | 0.392 |  | 0.239 |  | 0.381 |  | 0.847 |  |
| **C9 PS** |  |  | 12.7 |  | -14.1 |  | 32.6 |  | 4.7 |  | -3.1 |  |
|  |  |  | -35.8 | 61.2 | -37.3 | 9.0 | -34.2 | 99.4 | -25.2 | 34.6 | -43.7 | 37.4 |
|  |  |  | 0.608 |  | 0.232 |  | 0.338 |  | 0.757 |  | 0.880 |  |
| **C9 S** |  |  |  |  | -26.8 |  | 19.9 |  | -8.0 |  | -15.8 |  |
|  |  |  |  |  | -71.0 | 17.2 | -55.2 | 95.0 | -58.1 | 42.2 | -70.0 | 38.4 |
|  |  |  |  |  | 0.234 |  | 0.603 |  | 0.755 |  | 0.568 |  |
| **GRN PS** |  |  |  |  |  |  | 46.8 |  | 18.9 |  | 11.0 |  |
|  |  |  |  |  |  |  | -16.7 | 110.3 | -6.1 | 43.8 | -24.2 | 46.3 |
|  |  |  |  |  |  |  | 0.149 |  | 0.138 |  | 0.540 |  |
| **GRN S** |  |  |  |  |  |  |  |  | -27.9 |  | -35.7 |  |
|  |  |  |  |  |  |  |  |  | -96.1 | 40.3 | -106.0 | 34.5 |
|  |  |  |  |  |  |  |  |  | 0.423 |  | 0.318 |  |
| **MAPT PS** |  |  |  |  |  |  |  |  |  |  | -7.8 |  |
|  |  |  |  |  |  |  |  |  |  |  | -50.1 | 34.4 |
|  |  |  |  |  |  |  |  |  |  |  | 0.716 |  |
| **MAPT S** |  |  |  |  |  |  |  |  |  |  |  |  |
|  |  |  |  |  |  |  |  |  |  |  |  |  |
|  |  |  |  |  |  |  |  |  |  |  |  |  |

 

**c)**

|  | **C9 PS** |  | **C9 S** |  | **GRN PS** |  | **GRN S** |  | **MAPT PS** |  | **MAPT S** |  |
| --- | --- | --- | --- | --- | --- | --- | --- | --- | --- | --- | --- | --- |
| **Controls** | 2137.2 |  | 2302.5 |  | 480.5 |  | **3683.5** |  | 482.8 |  | 3470.7 |  |
|  | -2411.8 | 6686.1 | -2698.7 | 7303.6 | -521.5 | 1482.4 | **776.5** | **6590.5** | -868.6 | 1834.2 | -762.8 | 7704.3 |
|  | 0.357 |  | 0.367 |  | 0.347 |  | **0.013** |  | 0.484 |  | 0.108 |  |
| **C9 PS** |  |  | 165.3 |  | -1656.7 |  | 1546.4 |  | -1654.4 |  | 1333.6 |  |
|  |  |  | -6694.5 | 7025.1 | -6379.8 | 3066.4 | -3585.2 | 6677.9 | -6157.0 | 2848.3 | -5142.6 | 7809.8 |
|  |  |  | 0.962 |  | 0.492 |  | 0.555 |  | 0.471 |  | 0.687 |  |
| **C9 S** |  |  |  |  | -1822.0 |  | 1381.1 |  | -1819.6 |  | 1168.3 |  |
|  |  |  |  |  | -6874.8 | 3230.8 | -4045.3 | 6807.5 | -7088.9 | 3449.6 | -4972.6 | 7309.1 |
|  |  |  |  |  | 0.480 |  | 0.618 |  | 0.499 |  | 0.709 |  |
| **GRN PS** |  |  |  |  |  |  | **3203.1** |  | 2.3 |  | 2990.3 |  |
|  |  |  |  |  |  |  | **10.3** | **6395.8** | -1622.0 | 1626.7 | -1401.6 | 7382.1 |
|  |  |  |  |  |  |  | **0.049** |  | 0.998 |  | 0.182 |  |
| **GRN S** |  |  |  |  |  |  |  |  | **-3200.7** |  | -212.8 |  |
|  |  |  |  |  |  |  |  |  | **-6381.2** | **-20.2** | -5264.0 | 4838.5 |
|  |  |  |  |  |  |  |  |  | **0.049** |  | 0.934 |  |
| **MAPT PS** |  |  |  |  |  |  |  |  |  |  | 2987.9 |  |
|  |  |  |  |  |  |  |  |  |  |  | -1458.9 | 7434.8 |
|  |  |  |  |  |  |  |  |  |  |  | 0.188 |  |
| **MAPT S** |  |  |  |  |  |  |  |  |  |  |  |  |
|  |  |  |  |  |  |  |  |  |  |  |  |  |
|  |  |  |  |  |  |  |  |  |  |  |  |  |

 

**Supplementary Table 2: Mean (standard deviation) concentrations of the microglial activation markers in each decade of life within the controls (excluding the 2 undetectable concentrations of CHIT1 in controls). Spearman correlation of each measure with age was as follows: TREM2 r=0.42, p=0.0008, YKL-40 r=0.71, p<0.0001, CHIT1 r=0.21, p=0.1013.**

| **Age group** | **Number of participants** | **TREM2** **(pg/mL)** | **YKL-40** **(ng/mL)** | **CHIT1** **(pg/mL)** |
| --- | --- | --- | --- | --- |
| **20-29** | 8 | 3022.2 (1119.9) | 63.8 (16.6) | 1104.6 (845.1) |
| **30-39** | 13 | 3260.7 (2185.5) | 71.3 (23.0) | 979.0 (545.9) |
| **40-49** | 17 | 3453.5 (1423.0) | 91.6 (26.0) | 1195.9 (1435.9) |
| **50-59** | 13 | 4508.3 (3267.4) | 117.8 (48.3) | 1317.5 (905.1) |
| **60-69** | 11 | 5914.4 (1250.5) | 186.6 (99.2) | 2086.6 (2411.3) |

**Supplementary Figure 1 Partial correlations (adjusting for age) of CHIT1 with MMSE in GRN mutation carriers a) presymptomatic, b) symptomatic**

**a)**

**b)**
